# The Parent–Child Patient Unit (PCPU): Evidence-Based Patient Room Design and Parental Distress in Pediatric Cancer Centers

**DOI:** 10.3390/ijerph18199993

**Published:** 2021-09-23

**Authors:** Tanja C. Vollmer, Gemma Koppen

**Affiliations:** 1 Architectural Psychology and Health, Faculty of Architecture, Technical University of Munich, Arcisstrasse 21, 80333 Munich, Germany; 2 Kopvol architecture & psychology, Mathenesserdijk 396, GV3026 Rotterdam, The Netherlands; gkoppen@kopvol.com

**Keywords:** architectural psychology, evidence-based design, hospital design, design criteria, hospitalization, parent–child interaction, parental health, child well-being, privacy, oncology

## Abstract

Children with cancer are frequently hospitalized during diagnosis and treatment. Since the early 1980s, parents are co-admitted because their presence positively affects children’s adjustment to hospitalization and reduces post-traumatic stress. However, the size and overall architectural design of the rooms were never adapted to the doubling of the occupancy rate. Since studies show that many parents experience high levels of distress due to their child’s illness, the purpose of this study was to investigate the impact of the architecture of the aged patient rooms on parental distress. A video observation targeted parent–child interaction related to five architectural determinants: (a) function and place of interaction, (b) distance between parent and child, (c) used space, (d) withdrawal, and (e) duration of the interaction. A total of 22 families were included in two Dutch children’s hospitals. Results show a significant association between parental distress and three architectural determinants: The less anxious the parents were and the better they estimated their child’s well-being, the more distance they created between themselves and their child, and the more space, privacy, and withdrawal options were used. These findings are discussed within a new patient room typology, the parent–child patient unit (PCPU), which reacts to the evident association of parental distress and the design.

## 1. Introduction

Children and adolescents with cancer are frequently hospitalized during diagnosis and the course of treatment. In one-third of the cases hospitalization can last several weeks or add up to several months within a year due to complications [1,2,3]. Since the early 1980s, parents in Europe are allowed to stay overnight. During this time, it was scientifically proven that parents are the primary source of psychosocial support for their children in the hospital [4] and that their presence has a positive effect on children’s adjustment to hospitalization [5]. While the occupancy rate of the patient rooms doubled due to the co-admission of parents, called rooming-in, the size and overall design of the rooms did not change. Despite the decades-long practice of rooming-in, there is no evidence on the impact of the architectural design of the outdated patient rooms on parental distress. Therefore, the aim of the study was to investigate the relationship between patient room design and parental distress during hospitalization, using a theoretical framework of architectural determinants [6,7,8].

### 1.1. The Psychological Impact of Childhood Cancer

In Europe, 35,000 children are diagnosed with cancer every year [9]. Diseases such as childhood cancer that were once fatal are now successfully treated, and the survival rate is much higher than 20 to 30 years ago [10]. Improvements in therapeutic possibilities and early detection have led to a survival rate of more than 75% [11]. The challenge of this positive development is to manage the serious side effects that many survivors experience. Mortality has often been replaced by lifelong morbidity [10]. This morbidity and its treatment significantly confront children, adolescents, and their parents with chronic stress, which can contribute to emotional and behavioral problems [12]. In addition, emotional well-being and behavior are also influenced by the primary therapies and surgeries that can affect children’s language and cognitive performance [13]. Even with a good prognosis, the family and child feel threatened with death when given the diagnosis [14]. Chemotherapy, in particular, promotes a series of visual and traumatic transformations, for example, apathy, loss of appetite, weight loss, alopecia, mouth bleeds, nausea, vomiting, and diarrhea [15]. In this experience, children and parents live with feelings of sadness, fear, anxiety, and depression [12]. Qualitative analysis of deepening interviews defines four major themes of distress: (a) loneliness, isolation, and loss of a normal childhood; (b) decreased enjoyment of food; (c) physical discomfort and disability; (d) emotional responses to cancer, specifically anger and fear [15].

### 1.2. The Relation between Childhood Cancer and Parental Distress

Studies indicate that many parents experience high levels of anxiety, depressiveness, and uncertainty [16,17] when their children are seriously ill [18,19,20]. Parents of children with cancer experience high levels of psychological and social distress [21,22]. High parental distress is a barrier to effective participation in child care and can adversely affect the hospitalized child [23]. It has also been associated with negative long-term adjustment for parents and children [21,24,25,26,27]. Research regarding the long-term effects of hospital-related stress on family members has predominantly focused on relatives of patients with life-threatening or life-limiting illnesses. A recent review of the literature suggests that a significant number (30–70%) of family members of intensive care unit patients experience symptomatic anxiety and depression, or post-traumatic stress symptoms (PTSSs) for months or years afterward [28]. The prevalence of symptoms is estimated to be 10–40% in parents of children with potentially life-threatening conditions [26,29,30]. The degree of anxiety and depression is not routinely assessed, and hospital personnel often do not recognize it [31].

### 1.3. The Psychological Consequences of Hospitalization and the Birth of Rooming-In

In addition to physical and emotional distress, prolonged time in a hospital setting can deprive children of social and family relationships, prevent the formation of peer groups, and hinder independent activities that are essential to healthy child development [32,33]. Children who are medically and socially fragile are known to have worse outcomes than children with only one of these conditions [34]. Separation from parents is one of the greatest stressors for hospitalized children and adolescents [35,36,37], which is measurable in elevated temperature, pulse rate, blood pressure, post-operative emesis, disturbed sleep, and an extended period of recovery [38]. Parental presence with a well-supported parent is therefore the most effective coping intervention [4]. Young children are especially stressed by this separation because parents typically protect them from the danger of daily life [39]. Additionally, parents of hospitalized children are often overwhelmed with anxiety or uncertainty of their role, which interferes with the emotional support their children need [40] and therefore leads to even greater stress and confusion for children [41,42]. Regression, sadness, separation anxiety, apathy or withdrawal, fears of the dark, and sleep disturbances are behavioral and emotional changes in children that manifest during hospitalization and can persist for weeks or months after discharge [43,44]. The quality of the parent–child relationship plays a crucial role in the psychological development of children [45,46,47]. According to different theories, the emotional bond between the parent and child is considered the most important dimension [46,47,48]. Furthermore, it is a natural desire of parents to participate in their child’s care and emotional and practical support [23]. First models show that the role of parents’ coping may be important to consider on several levels as parents may serve as a resource to support children’s coping [12]. On the basis of these models and the proven facts that social isolation and family separation have a negative impact on hospitalized children and their families, the concept of parental co-admission, also called parental rooming-in, has emerged. 

### 1.4. The Architectural Consequences of Rooming-In and Its Relationship to the Psychosocial Distress of Parent and Child

Parental co-admission may answer to children’s needs of emotional support and normality and also the parents’ needs for receiving adequate information [49,50,51]. A majority of children and adolescents report worrying about the unfamiliar environment, of not feeling safe [51], and would like someone to stay overnight in the hospital during medically difficult times, with the mother mostly being preferred [52]. Parents chose to participate because of concern for the child’s emotional welfare [53]. A study by Smith, Hefley, and Anand [54] showed that stress levels of parents with parent bed spaces on an intensive care unit are lower than of parents who have to leave the hospital overnight. Tandberg et al. [55] compared levels of depression, anxiety, stress, and attachment scores among parents in a single-family room unit vs. an open bay unit. Mothers of single-family rooms had significantly lower depression scores, compared to those in the open bay unit and were significantly considered less as being at high risk for depression (14% vs. 52%). Both mothers and fathers reported significantly lower stress levels during hospitalization.

On the other hand, there is a critical perspective on parental co-admission referring to a potential negative impact on children’s autonomy development [5]: One of the core dimensions of parenting is the promotion of autonomy for children and adolescents [45,56]. Low levels of autonomy lead to poor child adjustment and health outcomes [57]. This might be moderated by further factors such as the age of the hospitalized child [39] and the severity of the illness. Parents who are staying at the bedside of their child have the desire to be involved in their care but simultaneously recognize that they have significantly less control over their child [58]. This could lead to increased stress levels of parents. In addition, Franck et al. [59] found that those who sleep at their hospitalized child’s bedside experience more sleep disruption, report poorer sleep quality, and feel less rested, compared to parents sleeping at hospital’s onsite spaces. Overall Franck et al. [60] highlighted that research is urgently needed to better understand the needs and preferences in order of family accommodation during a child’s hospitalization.

The hospital’s physical environment is an essential component of the care provided in children’s health care settings and plays an important role in supporting the practice of family-centered care [61]. Evidence exists that the hospital environment can influence the amount and degree of interaction that occurs [62]. A thoughtfully designed hospital environment can support patients and families psychologically by providing greater control, protecting privacy, and facilitating communication and participation in care [63]. Social support can positively influence children’s coping and health care outcomes during hospitalization [4,64,65]. Studies indicate that contact with peers results in significant benefits in social and communication skills and in the development of greater self-confidence and independence among pediatric patients [66,67]. Peer-to-peer interaction is particularly important for hospitalized adolescents [68,69].

Parents seem to benefit from interactions with other parents sharing similar experiences [70]. Parent-to-parent support provides parents with information, emotional support, a sense of being understood, friendship, mentoring, role modeling, assistance with problem solving, and a base for advocacy efforts [71]. Support from other parents is more likely to be accepted than formal support [72]. Parents have cited support from other parents as an essential factor in helping them cope successfully with the stress of a hospital experience [64]. Parents who are supported are better able to help their child with cancer to cope with the stress of illness, treatment, and hospitalization, particularly in middle infancy [73].

The architectural beginning of rooming-in was made with a study undertaken at the James Whitcomb Riley Hospital for Children in Indianapolis with the so-called parent care pavilion (PCP) [74]. Admitted to the PCP, parents gained an understanding of the child’s problem over time, rather than in one final hurried conference with the doctor. Upon discharge, mothers were evidently coping well. Since one of the benefits of the pavilion was the elimination of psychological trauma associated with hospitalization [75], it became a worldwide role model of co-admitting parents. The downside of this development was the lack of adapting new hospitals to the whole PCP concept, for example, clustering patient rooms to a family pavilion with a play and a dining area, or providing only one-bed hospital rooms with twin beds for parents and the adequate space, and providing (at least) a curtain to screen-off parent’s sleeping area, which was the design proposal in the PCP. Furthermore, the PCP was neither advised for severely ill or dying children, nor for patients, who require intravenous fluids or 24 h monitoring [74]. This was due to the pure care character of the PCP, which did not function as a cure hospital of high-performance medicine, as they have been built since the 1980s.

## 2. Materials and Methods

### 2.1. Procedures 

This paper reports a non-published dataset of the additional findings of the Child Development Supportive Building Project, a prospective cohort study of parental and child distress and spatial awareness during admission to a Dutch pediatric oncology ward [76]. This study was carried out in several phases and lasted a total of 2 years, partly because it was directly linked to new building projects. Each day of the data collection phase, a member of the research team identified all new and expected admissions on each ward and gave eligible families written information about the study. The researcher discussed the study with the child’s parent and if both wanted to participate and obtained written consent and initial questionnaires from them. Parent participants completed the stress-related questionnaires every third day during hospital admission. A video recording system was used to enable the continuous record of parent–child interaction and behavior in the patient room. The system was switched off during the nights to guarantee privacy.

Demographic and clinical information was collected from a short questionnaire: sex of the parent, relationship status of the parent, age of the child, diagnosis of the child, and previous hospital experience of the family. Parents were also asked questions about the distance and travel time between the family’s home and hospital. Inclusion criteria were all parents co-admitted to the wards during the total stay of their child and using the rooming-in function during the total admission period. Exclusion criteria were parents hospitalized for less than seven nights. After contacting 26 parents, 22 were included in the study. All 22 parents came from different families. Of those, 9 parents were recruited at the study site in Amsterdam, and 13 parents at the study site in Rotterdam.

### 2.2. Measures

#### 2.2.1. Behavioral Observation and Its Associated Architectural Determinants

A data collection method with structured video observations was used targeting parent–child interaction while being together in one patient room. The observation captured five architectural determinants as follows: (a) function and place of interaction, (b) distance between parent and child, (c) used space for interaction, (d) used possibilities for withdrawal, and (e) duration of the interaction. The study was carried out in two pediatric oncology wards in The Netherlands: One located in the Sophia Children’s Clinic of the Erasmus Medical University Hospital in Rotterdam and the other located in the Emma Children’s Clinic of the Amsterdam University Medical Center. 

#### 2.2.2. Well-Being of the Child

The well-being of the child was daily scored by parents on a visual analog scale, which could be read out in three categories: 1 = good (J), 2 = medium (K), and 3 = bad (L). Based on the international research literature described in the introduction, the well-being of the child was treated as a factor of parental distress.

#### 2.2.3. Hospital Anxiety and Depression Scale (HADS)

HADS is a 14-item questionnaire with 2 subscales measuring anxiety and depression. Each subscale has 7 items and total scores range from 0 to 21. Scores of 0–7 are considered normal, with 8–10 indicating mild, 11–14 moderate, and 15–21 severe levels of anxiety or depression [77]. It has demonstrated validity and reliability [78] with Cronbach’s alpha of 0.81 for both the anxiety and depression subscales.

#### 2.2.4. Parental Perception of Uncertainty in Illness (PPUS)

The PPUS is a 31-item questionnaire developed by Mishel [79] and assesses parental appraisal of uncertainty in relation to their child’s illness. Total scores range from 0 to 155 with higher scores indicating greater uncertainty [16]. There are four subscales: (1) ambiguity, (2) lack of clarity, (3) lack of information, and (4) unpredictability. The scale has demonstrated good validity and reliability [79,80,81,82].

#### 2.2.5. Statistics 

Quantitative data were summarized using IBM SPSS Statistics, Version 27. Descriptive statistics were computed for all variables. A series of correlations were conducted to test the associations between parental stress and parental behavior related to parent–child interaction and the architectural design of the patient rooms while controlling for relevant demographic, hospitalization-related, and other factors.

## 3. Results

### 3.1. Respondents Characteristics 

Descriptive and health-related characteristics are given in Table 1. Among 22 respondents, 12 were female (55% mothers), and 10 were male (45% fathers). Most of them (86%) were in an active relationship, 9% were single parents, and 5% were divorced or widowed. Almost half of the children (45%) were older than 5 years, whereas 40% were younger than 3, and 15% were between 3 and 5 years old. With 36%, leukemia was the most present diagnosis of the children, followed by non-Hodgkin lymphoma, with 18%. 12 families lived in the city where the hospital was located, 9 in suburbia, and 1 family lived more than 50 km away from the hospital. More than half of the families (60%) experienced their first stay in the hospital.

### 3.2. Patient Room Architecture

**Figure 1:**
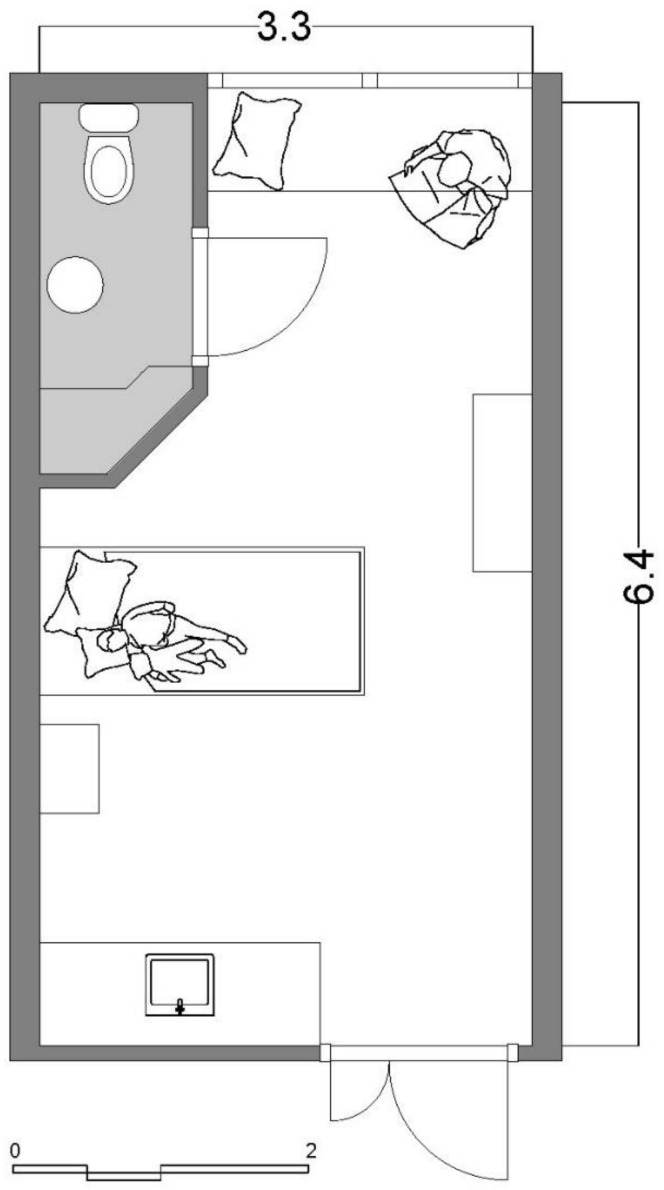
Average patient room typology of the observed hospitals. Net m^2^: 18.8 m^2^ patient room and 2.4 m^2^ bathroom.

Figure 1 shows the average patient room typology of the two observed Dutch hospitals. The room is 6.4 m long and 3.3 m wide. The layout is a rectangular single-bed room with a direct view from the entrance to the patient bed. The entrance door is located opposite a window that could not be opened because of safety reasons. The room includes a small bathroom either directly at the door side or at the window side, as shown in Figure 1. The parent’s bed is located under the window, is directly visible from the entrance, and functions as a couch throughout the day. The room includes a small cupboard that also functions as a writing desk for doctors and nurses and a hand wash cabinet with medical materials inside. Except for the bedside cabinet of the child, there is no other table in the room.

### 3.3. Parent–Child Interaction and Its Relationship to Architectural Determinants 

**Figure 2:**
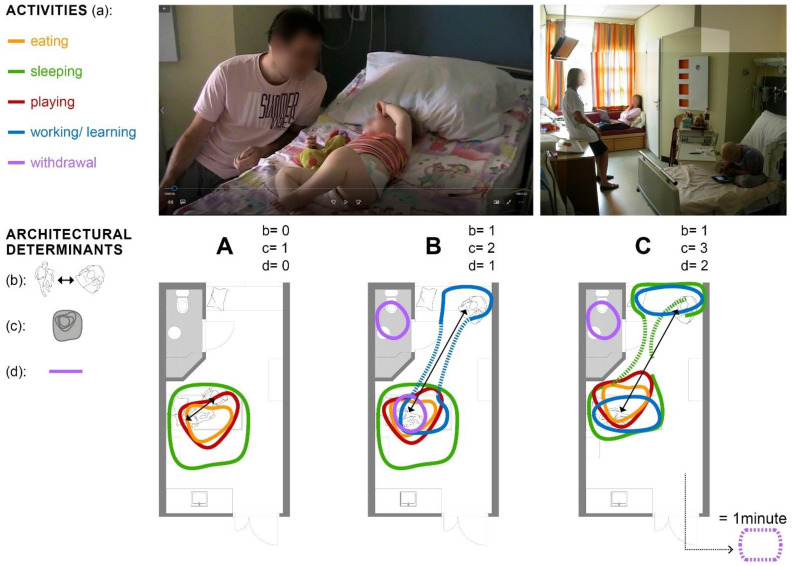
Behavioral observation analysis: (**A**) distance between parent and child; (**B**) used space for interaction; (**C**) used possibilities for withdrawal.

With a minimal exception of half an hour per day, parents and the child reside together in their room 24/7 (e). Analysis of the behavioral observations shows that the patient room is used for the entire daily routine of parent and child and all related functions—namely, (a) eating, sleeping, playing, learning (child), working (parent), and withdrawing. The place where these functional activities occur varies from time to time. Figure 2 shows that analysis of behavioral observation generally reveals three situations ((A), (B), (C)) that differ in three architectural determinants ((b), (c), (d)): the distance between parent and child (b), the space used for interaction (c), and the used means of withdrawal (d). For the analysis of the relationship between interaction and architectural determinants, the functions observed as outcomes (a) (eating, sleeping, etc.) are considered in relation to the use of space by parents and children. In order to better distinguish the individual functions, they are each given a different colored line in Figure 2 and are referred to as “activities”. Analysis reveals that in situation (A), within a day, only the activities of eating, sleeping, and playing occur. All activities are performed through the interaction between parent and child. There is no real spatial distance, but the child’s bed is the central place of action and even residence for both persons. In situation (B), the activities of learning or working, and withdrawing additionally occur, without leaving the patient room. The parent maintains a greater distance from the child, and the only spatial withdrawal occurs in the room-integrated bathroom. Only in situation (C), it can be observed that parent and child work and learn separately and that the parent sleeps on a separate bed. Only in this situation, the physical contact with the child is reduced to a minimum, and the parent leaves the room several times a day for a few minutes.

### 3.4. Parental Stress 

Measures of parental distress during hospitalization are presented in Table 2. More than half of the parents experienced moderate or severe anxiety (52% with scores > 10), and over a third (33% with scores > 10) experienced moderate or severe depression. The mean values at the three different measuring points (MP) did not significantly vary. The overall means are displayed in Table 2. Uncertainty was moderate and within the range of the literature reported values. The parents’ estimated well-being of the child was significantly different between the three measuring points. Mean values decreased from 2.8 (SD = 0.2) at MP1 to 1.5 (SD = 0.5) at MP3, which represents an increase in the subjective estimated well-being of the child over a time span of seven days.

### 3.5. Association of Parental Disstress and Architectural Determinants 

Table 3 shows the correlations between parental disstress and the architectural determinants (b), (c), and (d): anxiety is highly and significantly correlated with all three determinants. The less anxious the parent is, (b) the greater is the distance between parent and child (r = −0.360, *p* < 0.001), (c) the more space is used for parent–child interaction (r = −0.29, *p* < 0.001), and (d) the more withdrawal is sought (r = −0.297, *p* < 0.001). There is no significant correlation between depression and the architectural determinants of the patient room, whereas uncertainty is significantly correlated with two architectural determinants: The more certain the parent is about the medical and physical conditions of the child, (b) the greater is the distance between parent and child (r = 0.258, *p* < 0.05) and (c) the more space is used for parent–child interaction (r = 0.199, *p* < 0.05).

### 3.6. Association of Arental Stressor “Well-Being of the Child” and Architectural Determinants 

Table 4 shows the correlations between the parents’ estimated well-being of the child and the architectural determinants (b), (c), and (d). Child well-being is significantly correlated with all three determinants at all three measuring points: the better the child’s well-being is, (b) the greater is the distance between parent and child (MP 1: r = 0.360, *p* < 0.001), (c) the more space is used for parent–child interaction (MP 1: r = 0.292, *p* < 0.001), and (d) the more withdrawal is sought (MP 1: r = 0.291, *p* < 0.001).

**Figure 3:**
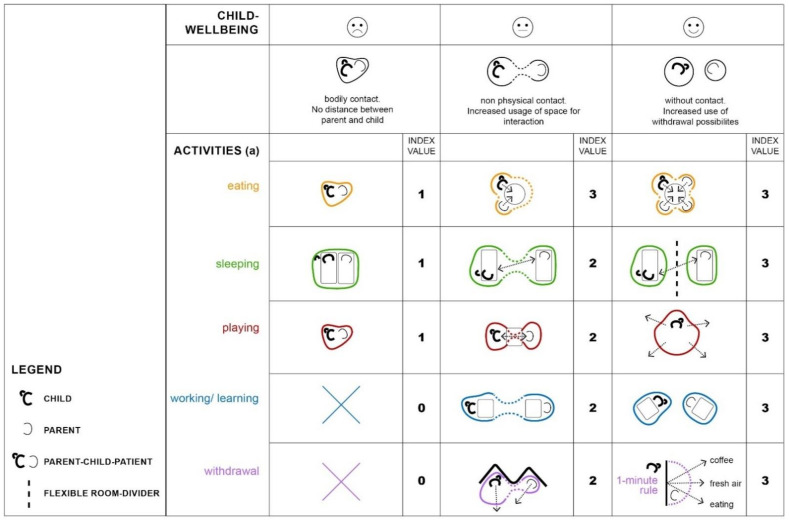
Graphical depiction of the relationship between child well-being and architectural determinants, divided into five daily activities.

Figure 3 graphically depicts the relationship between child well-being and architectural determinants. The detailed analysis of the behavioral observation shows that all daily activities reveal the correlation. The better the child’s health is according to the parents’ assessment, the further they move away from the child’s bedside. Interactions become fewer but the space that is used for it increases. Activities such as sleeping, learning, working, and eating occur in separate places within the room and sight. In some cases, room dividers such as self-made paravents are used to reinforce the spatial separation and thus the feeling of privacy. If the child is very well according to the parents’ estimation, they even leave the room for a few minutes several times a day.

## 4. Discussion

### 4.1. The Parent–Child Patient: A Negative Result of Patient Room Design

Although studies have well demonstrated for years that parents of critically ill children are extremely stressed and at high risk for secondary illnesses due to hospitalization, there has been no research on the impact of patient room architecture on parental stress. Our study indicates, for the first time, that there is a relationship between parents’ anxiety and uncertainty, as well as their assessed well-being of the child, and architectural determinants. Franck et al. [60] highlighted that research is urgently needed to better understand the needs and preferences in order of family accommodation during a child’s hospitalization. As one answer to this request, our results show that the better the child’s health condition is from the parents’ perspective, and the more certain parents become about their child’s condition, the less anxious they are, and the more distance between parent and child is established within the patient room. In addition, as the parents’ anxiety decreases and the child’s well-being increases, direct interaction is reduced, and withdrawal is sought. However, the room itself is left only with a few exceptions and only for a few minutes during the entire stay. These findings are consistent with studies in other fields that show a correlation between parental insecurity, decreasing trust, and increased closeness to the child. In these studies, both external factors, such as the rotation system of nursing staff [83], and internal factors, such as children’s speech, language, and communication problems [84], can be found to increase insecurity and the need for closeness between children with cancer and their co-admitted parent. Research by Cornwell et al. on voice disorders as a component of cerebellar tumor (CT) [85] complements a study by Gonçalves et al., who described the incidence of speech, language, and hearing difficulties and disorders in children and adolescents with central nervous system (CNS) tumors [13]. The authors noted that speech–language and hearing complaints and symptoms were reported by 42% of the patient sample and could be recognized as risks associated with pediatric cancer treatments.

Figure 1 shows that there is no way for parents to avoid the child’s demand for closeness and thus reduce their own stress. The outdated floor plans of the patient rooms force parents and the child to constantly be confronted with the stressors of one another and transfer their stresses. The effect sizes of the architectural determinants of the outdated patient rooms are too low to exert a measurable influence on the parents’ stress experience; that is, the distance created between parent and child does not, due to the design of the rooms, create a real optical or acoustic separation of parent and child, which could reduce anxiety and strengthen a sense of security. Parents observe every movement of their child even at a maximum distance in the rooms. This results in the continuous mutual transmission of fears and the disturbance of their own, even relaxing activities. Parents are disturbed in their sleep by every movement of the child or do not allow themselves to fall asleep in the first place. Th literature reveals that sleep disturbance is one of the main distressing factors while being hospitalized in general and is a special problem of rooming-in [86]. Furthermore, many children and adolescents with cancer experience a severe interruption to mealtime dynamics, which can have a significant impact on the emotional needs of families [87]. The outdated patient rooms, which we documented, do not meet these needs. There is no place or facility that supports the recreation of (eating, sleeping, or playing) normality. In conclusion, in the old patient rooms, no single architectural determinant contributes to supporting the psychosocial well-being of parents and the child. On the contrary, the space hinders the establishment of normal and healthy interaction, on the one hand, and sufficient privacy on the other. It, therefore, becomes a stressor.

**Figure 4:**
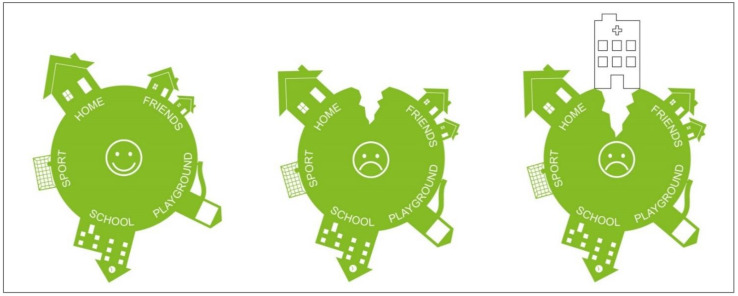
Graphical depiction of the emergence of the parent–child patient [90].

This result explains that despite the observed tendency of parents to withdraw when the child is better, there is no decrease in their anxiety and stress levels. Numerous studies have already documented the high stress levels of parents, who were hospitalized in these kinds of outdated rooms [58,88]. One-third of them even developed post-traumatic stress disorder [16]. Furthermore, Franck et al. showed that housing families outside the hospital have advantages over rooming-in in terms of parental well-being [89]. Unlike in this study, the parents in our study are not willing to leave the room for more than a few minutes. The observed unwillingness to leave the room for longer periods of time confirms the constant emotional alertness of the parents, which explains the constant fear and tension. We have previously described this phenomenon as the parent–child patient [76,90]. Figure 4 describes the emergence of this parent–child patient: When children become seriously or life-threateningly ill, their safe world is suddenly “cracked”, and illness becomes the focus of their life. Rodriguez et al. [91] asked 106 children with cancer and their parents to report on cancer-related stressors for the child near the time of diagnosis: Daily role stressors included missing school days or falling behind in schoolwork, not being able to accomplish the things they used to, having to go to the hospital or clinic visits, and concerns about family and friends. The stressors related to cancer treatment involved feeling sick or nauseous from treatments, concerns about changes in appearance, and pain and soreness from medical procedures. Uncertainty about cancer included stress related to not understanding what doctors discuss about cancer, feeling confused about what cancer is and its causes, and concerns about the future. Based on both children’s and parents’ reports, all three types of stress were experienced as moderately to highly stressful and with relatively high frequency for children [91].

**Figure 5:**
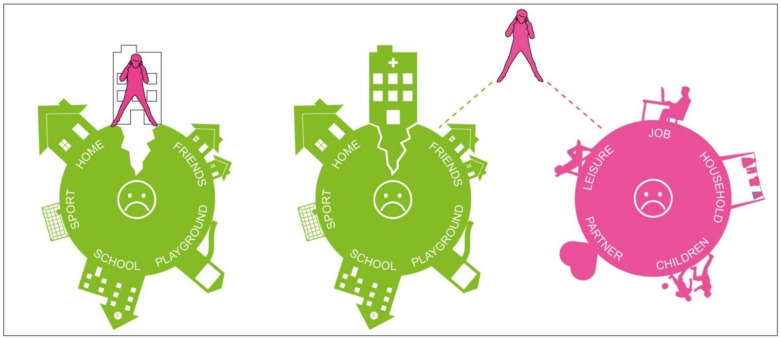
Graphical depiction of the so-called parenting balancing act [90].

If children are admitted to the hospital, the crack becomes larger, since the known living environment—the child’s normality—is suddenly reduced to that of the hospital. Moreover, this environment is initially associated primarily with pain, fear, and illness. In order to adapt to this environment, the parent admitted to the hospital acts as a “healing bridge”, shown in Figure 5. While studies have already shown that this “healing bridge” is successful and that the co-admission of the parents leads to a reduction in the children’s anxiety and psychological stress, the parents have little opportunity to reduce their own anxiety and stress. On the contrary, in addition to life in the hospital, home life must also be mastered. Partnership, family, job, and siblings place additional demands that lead to the so-called parenting balancing act during this time [76,88]. Each phase of a chronic illness can present children and their families with significant challenges and stressors. However, there is evidence that chronic conditions may exert greater psychological and physical stress than acute illnesses that resolve quickly [92]. This is consistent with more general models of the adverse effects of chronic stress as a consequence of processes of allostatic load that include the physical and psychological wear and tear associated with prolonged or repeated demands that characterize chronic stress [93]. In a recent study of parents residing in a Ronald McDonald House during their child’s hospitalization, family functioning mediated the relationship between family hardiness and caregiver anxiety, with both family functioning and hardiness reducing anxiety during the hospitalization [20]. Distance from the hospital has also been shown to affect family function when children have chronic conditions [94].

### 4.2. The Parent–Child Patient Unit (PCPU): A Consequent Architectural Approach Based on the Relationship of Patient Room Design and Parental Distress

The hospital design literature depicts many examples of the ways in which hospital design can provide opportunities for both privacy and social interaction and uphold principles of family-centered care, but patient rooms that meet the parents’ needs to be close to their children and at the same time being supported in finding relaxation, privacy, and withdrawal are missing. Despite the decades-long practice of encouraging parents to take an active part in the care of their hospitalized child, there is little evidence on how to accommodate them best, in a way that enables them to be effective and healthy participants in their child’s treatment and recovery [60].

For example, as our results—and many others—show, there is a high demand for privacy, even related to the described parent–child patient. Privacy is directly related to control issues. As such, privacy can be defined as the selective control of social interactions [95,96]. Children and their families want to control to whom they are accessible. This ability to control interactions is so important that these skills may be even more important than the interaction itself [4]. A sense of control is related to opportunities to modify or alter aspects of the environment [97,98]. In hospitals, the established routine render parents helpless. In the outdated patient rooms, parents experience a loss of control related to almost every aspect of their daily lives: what and when to eat and when to receive visitors; they have little opportunity to leave the ward or patient room, are limited in their range of activities, and do not have control over their surrounding physical environment. Opportunities to exert control over the physical environment help combat the sense of helplessness. To counter the feeling of helplessness, Huisman et al. [99] recommended what they called “self-supporting systems” to enable hospitalized people to control many aspects of the room [100]. Ulrich’s theory of supportive design conceptualizes the ways in which the healthcare physical–social environment affects patients’ well-being, including the reduction of stress [101]. Ulrich proposed that physical and social healthcare environments promote well-being if they are designed to foster (a) a sense of control over physical–social surroundings, (b) access to social support, and (c) access to positive distractions.

**Figure 6:**
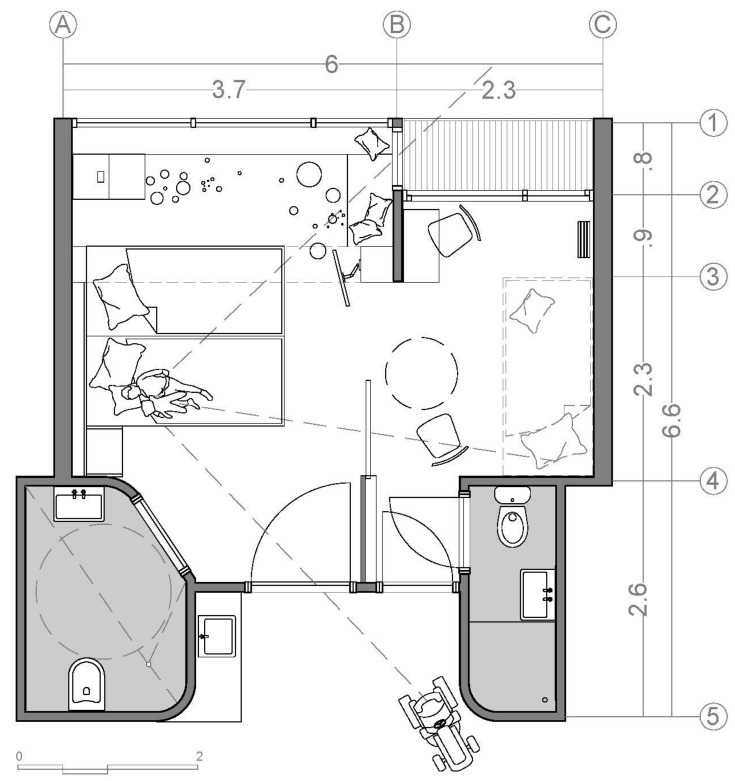
Floorplan of the Parent–Child Patient Unit (PCPU).

In 2010, the initiators of the Princess Maxima Center for Pediatric Oncology (PMC)—formerly known as the National Child Oncology Center (NKOC)—commissioned the development of design criteria that address these special needs of children with cancer and their co-admitted parents. The research and development project resulted in a novel patient room typology: the Parent–Child Patient Unit (PCPU) [76]. The PMC opened its doors in 2018 in Utrecht, The Netherlands. The PCPU has the following design criteria [76], which are graphically shown in Figure 6:

1. The patient room is divided into a child part and a parent part;
2. Both parts can be separated acoustically and/or visually and gradually, for example, by a sliding door;
3. Both parts have their own entrance and bathroom, as well as their own work or play and dining table;
4. Parents have a view of their child from the bed when the door is open;
5. The child part is clearly zoned into an entrance zone for medical and nursing activities and a private (play) zone where these activities do not occur;
6. The parent part has direct access to an outdoor area, such as a terrace or balcony;
7. The PCPU is embedded in functional services for parents within a perceived walking distance of one minute (1 min rule).

**Figure 7:**
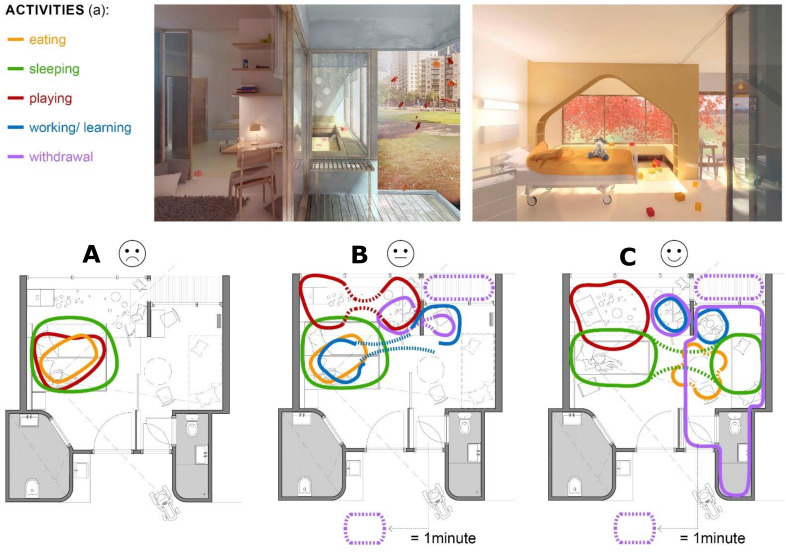
Graphical depiction of how the PCPU reacts to the association of parental distress and the three architectural determinants: (**A**) distance between parent and child; (**B**) used space for interaction; (**C**) used possibilities for withdrawal.

Figure 7 shows graphically how the PCPU reacts to the findings of this study: In the case of the negative well-being of the child, parents can sit and sleep close to the child and make the parent part available to another member of the family. In a potentially unfamiliar and stressful environment, the social support of others can ameliorate stress [102]. Social support is widely acknowledged as a psychosocial factor that influences health outcomes. In the case of medium well-being of the child, the parent can move to its own part of the room and fully control its own privacy by partly opening or closing the door. In the case of the positive well-being of the child, the two rooms can either be joined for activities with family and friends or separated to guarantee total privacy for both parent and child. In the latter case, the effect of positive distraction on hospitalized individuals can be fully achieved. In the model of patient-centered care, using positive distractions is emphasized [103,104]. The beneficial role of positive distraction in the health care environment is well documented [101,105,106,107,108,109,110]. Positive distractions help individuals to focus on stimuli other than their own discomfort and anxiety. These distractions include static stimuli such as reading material, photographs, and representational posters or paintings, as well as active stimuli such as music, companions, or contact with nature [111].

The PCPU provides temporary and gradual separation of parent and child, which evidently supports an active coping and autonomous development of the child. A number of dispositional coping strategies have been associated with parental mental health after a child’s diagnosis or hospitalization for a life-threatening illness. In general, avoidant or dissociative coping strategies are generally thought to be positively associated with PTSS [12,30,112,113]. Active coping strategies have also been previously identified as adaptive when coping with chronic illness and hospitalization [114]. Sung and Herbst considered that a key ethical priority in the consideration of harm for adolescents who spend more than a month in a hospital is to respect and foster autonomy [115]. Young—and maybe neurodevelopmentally compromised—children fully depend on their parents to establish treatment goals and decide whether burdensome medical therapies should be performed. Diekema [116] argued that parents’ decisions for their children need not be held to the best interest standard; instead, interventions should not cause undue harm. A temporary spatial separation, as defined for the PCPU, can help to reflect these central ethical considerations. 

Moran [117] discovered that hotel-like elements that emphasize hospitality and comfort could reduce anxiety and promote healing. The ambiance should be as personal as possible such as a homelike environment. Similarly, the effect of furniture arrangements could promote improvements in the wards’ psychosocial atmosphere, as reported by Baldwin [118]. Since there are no medical actions in the parent part of the PCPU, this part can be designed in a very personal and atmospheric way. When both parts are combined, the atmosphere determines the entire patient room and, even in situations where the child’s health is very poor, creates a health-supportive environment for the parent–child patient.

## 5. Conclusions

The study shows that the outdated patient room typology in (cancer) children’s hospitals does not do justice to the co-hospitalization of parents that has been prevalent in Europe since the 1980s. On the contrary, the room architecture obviously increases the anxiety, insecurity, and stress of parents and thus endangers their health. It creates the so-called parent–child patient and as such also negatively affects the well-being and recovery of the children.

The PCPU is a novel patient room typology as a response to the evidence provided in this study—namely, that three architectural determinants are significantly associated with parental stress during hospitalization: (1) distance between parent and child, (2) space for interaction, and (3) opportunities for withdrawal. As these determinants drive design decisions about the PCPU, it is possible to speak of an evidence-based architectural concept. 

The knowledge developed and concept presented in this study can help architects and decision makers in hospitals to create future inpatient environments that have a positive impact on the health of hospitalized parents and their children. At the same time, the study informs healthcare professionals, physicians, nurses, and therapists about the impact of patient rooms on their entrusted patients and provides science-based arguments for stress-reducing patient room design in pediatric oncology. The applicability of the data to other long-term hospitalized groups should be explored in further studies.

A major limitation of the study is the relatively small sample size, which is usually much higher for psychometric testing. However, because, to the best of the authors’ knowledge, no such studies have been conducted to date, the data presented provide a good initial indication of the associations examined between parental stress and patient room architecture and thus a basis for further research. In particular, the methodological approach demonstrated in this study may help other researchers in architectural psychology to integrate scientifically developed assumptions into a new building project and thus create testable architectural interventions in the first place. Particularly in terms of a post-occupancy evaluation of the PCPU, further research is needed.

## 6. Patents

The PCPU is a new patient room typology based on the architectural design concept developed by Kopvol architecture and psychology [76]. The concept is called OKE (ouder-kind-eenhed) in the Dutch original and was developed for the Princess Maxima Center of Pediatric Oncology, Utrecht—formerly known as the National Child Oncology Center (NKOC). Everyone is free to reuse the published material—including the architectural concept—if proper accreditation/citation of the original publication is given. 

## Figures and Tables

**Table 1:** Descriptive characteristics of the sample (N = 22).

Characteristics	N	%
Respondent		
Mother	12	55%
Father	10	45%
Relationship status		
In a relationship	19	86%
Single parent	2	9%
Divorced/widowed	1	5%
Age of child		
0–2	9	40%
3–5	3	15%
>5	10	45%
Medical diagnosis of child		
Langerhans cell histiocytosis	2	9%
Leukemia [All]	8	36%
Non-Hodgkin lymphoma	4	18%
Glioblastoma	1	5%
Brain tumor	2	9%
Neuroblastoma	2	9%
Rhabdomyosarcoma	1	5%
Osteosarcoma	2	9%
Time interval to first diagnosis		
0–2 years	16	73%
3–5 years	5	22%
>5 years	1	5%
Distance from home		
In the city	12	55%
Suburbia up to 50 km from city	9	40%
>50 km from city	1	5%
Family first stay in hospital		
No	9	40%
Yes	13	60%

**Table 2:** Measures of parental distress during child hospitalization (N = 22).

Parental distress	Mean (SD) ^1^	Range	Scores > 10 (%) ^2^
Anxiety (HADS)	12.07 (4.11)	5–20	52%
Depression (HADS)	7.55 (4.13)	0–16	33%
Uncertainty (PPUS)	85.7 (13.4)	60–133	
Well-being of child (VAS)			
MP 1 (day 1)	2.8 ^3,^* (0.2)	1–3	
MP 2 (day 4)	2.2 ^3,^* (0.6)	1–3	
MP 3 (day 7)	1.5 ^3,^* (0.5)	1–3	

^1^ Means were calculated over three measuring points (MP) while hospitalization. ^2^ Scores higher than 10 indicate potential psychological morbidity as anxiety disorder or depression. ^3^ Significant mean difference: * *p* < 0.05.

**Table 3:** Correlations of parental stress and architectural determinants of the patient room.

	Architectural Determinants		
Parental Disstress	Distance between Parent and Child	Used Space for Interaction	Used Possibilities for Withdrawal
Anxiety of parents (HADS)	−0.360 ***	−0.290 ***	−0.297 ***
Depression of parents (HADS)	−0.167	0.172	0.142
Uncertainty of parents (PPUS)	0.258 *	0.199 *	0.021

Significant correlation coefficients: * *p* < 0.05, *** *p* < 0.001.

**Table 4:** Correlations of the estimated well-being of the child and architectural determinants of patient room across three measure points.

	Architectural Determinants		
Parental Stressor	Distance between Parent and Child	Used Space for Interaction	Used Possibilities for Withdrawal
Well-being of child (VAS)			
MP 1 (day 1)	0.360 ***	0.292 **	0.291 **
MP 2 (day 4)	0.177 *	0.372 ***	0.342 ***
MP 3 (day 7)	0.333 ***	0.352 ***	0.151 *

Significant correlation coefficients: * *p* < 0.05, ** *p* < 0.01, *** *p* < 0.001.
